# Approach to Analyze the Diversity of Myxobacteria in Soil by Semi-Nested PCR-Denaturing Gradient Gel Electrophoresis (DGGE) Based on Taxon-Specific Gene

**DOI:** 10.1371/journal.pone.0108877

**Published:** 2014-10-03

**Authors:** Baiyuan Li, Qing Yao, Honghui Zhu

**Affiliations:** 1 Guangdong Institute of Microbiology, State Key Laboratory of Applied Microbiology Southern China, Guangdong Provincial Key Laboratory of Microbial Culture Collection and Application, Guangzhou, China; 2 Key Laboratory of Tropical Marine Bio-resources and Ecology, South China Sea Institute of Oceanology, Chinese Academy of Sciences, Guangzhou, China; 3 University of Chinese Academy of Sciences, Beijing, China; 4 College of Horticulture, South China Agricultural University, Guangzhou, China; University of Camerino, Italy

## Abstract

The genotypic diversity of insoluble macromolecules degraded myxobacteria, provided an opportunity to discover new bacterial resources and find new ecological functions. In this study, we developed a semi-nested-PCR-denaturing gradient gel electrophoresis (DGGE) strategy to determine the presence and genotypic diversity of myxobacteria in soil. After two rounds of PCR with myxobacteria-specific primers, an 194 bp fragment of *mglA*, a key gene involved in gliding motility, suitable for DGGE was obtained. A large number of bands were observed in DGGE patterns, indicating diverse myxobacteria inhabiting in soils. Furthermore, sequencing and BLAST revealed that most of the bands belonged to the myxobacteria-group, and only three of the twenty-eight bands belonged to other group, i.e., *Deinococcus maricopensis*. The results verified that myxobacterial strains with discrepant sequence compositions of gene *mglA* could be discriminated by DGGE with myxobacteria-specific primers. Collectively, the developed semi-nested-PCR-DGGE strategy is a useful tool for studying the diversity of myxobacteria.

## Introduction

Myxobacteria are Gram-negative bacteria that are capable of multicellular, social behaviour [1], [2]. The distinctive feature of myxobacteria is that the vegetative cells aggregate into large mounds and then a matured fruiting body formed, by means of a peculiar gliding motility under starvation conditions [3], [4]. Myxobacteria play important roles in establishing ecological function, as result of gliding motility, complex life cycle, fruiting bodies formation, degrade insoluble macromolecules and the production of bioactive compounds. However, the spatial and temporal variability at a community level is less well known.

Myxobacteria were found nearly everywhere from Antarctica to the tropics, and from water bodies to terrestrial [5]. Isolation technologies for myxobacteria usually involve the incubation of natural samples on a selective solid medium, to allow for the formation of fruiting body [3], [4]. However, many myxobacteria species could not formed its fruiting body under current culture conditions and resulted in missing numerous strains. Until now, the recognized myxobacteria are only classified into three suborders, six families, 23 genera and approximately 50 species [6]–[9]. The works about the myxobacteria were very limited mainly due to insufficiency incubation strategy and limited strain information.

Myxobacteria are very common and ubiquitous organisms in soil. Dawid surveyed 1398 soil samples from 64 countries, and suggested that only 1∼10 myxobacterial species could be found in one soil sample [5]. It was estimated that 1 g of soil could contain 10^6^ or less cells of myxobacterial species [10]. However, these results are derived from the traditional culture-dependent techniques which suffering from many limited factors, resulted in an underestimation of the diversity and occurrences of myxobacteria [1], [5]. In this study, we developed a culture-independence method, PCR-denaturing gel electrophoresis (DGGE), to exploring the diversity of myxobacteria in soil. DGGE can distinguish the PCR amplicons with the same length according to the nucleotides composition. DGGE have been frequently applied to study the microbial community composition of different environmental samples, and Muyzer et al. firstly expanded it to study microbial genetic diversity [11], [12]. Nested PCR-based diagnostic assays were also successfully developed to determine the diversity of sulfate-reducing bacteria in complex microbial communities [13]. The *mglA* gene was selected as a putative target for DGGE analysis, because they are conserved and the only one solo gene could control the motility behavior of myxobacteria [14]. The aim of this paper was to develop a culture-independent method (DGGE) to explore the diversity of myxobacteria in complex microbial communities. The method developed in this study was evaluated by using pure cultures and by using soil samples. We confirm that this method can provide a more accurate strategy to study the distribution and discovery of myxobacteria in complex communities.

## Materials and Methods

### Soil sample collection

Soil samples were collected from the saline-alkaline soils of Xinjiang, China. No specific permits were required for the described field studies. We state that no specific permissions were required for the locations where the soils were sampled and we confirm that the location is not privately-owned or protected in any way and that the field studies did not involve endangered or protected species. Eight soil samples (S1∼S8) were collected by a sterile spatula from the upper layer (5∼15 cm), and the soil samples were air-dried immediately. Then the sample was transported to the laboratory and kept at 4°C for further analysis.

### Myxobacteria and other bacteria strains cultures

All cultures used in this study were obtained either from the DSMZ or GIMCC, a culture collection located in south China (Table 1). All myxobacterial strains were grown on VY/2 medium [15] at 30°C for 3∼5 days. The rest of the bacterial strains were grown on LB (Luria-Bertani) agar at 37°C for 1 day. Genomic DNAs isolated from these cultures were used as template to test the specificity of primers.

**Table 1:** Strains used in this study.

Strain	Isolate	Taxonomy	Source^a^
Myxobacteria			
*Myxococcus xanthus*	GIM1.685	Bacteria; Proteobacteria; Deltaproteobacteria; Myxococcales	GIMI china
*Myxococcus fulvus*	GIM1.686	Bacteria; Proteobacteria; Deltaproteobacteria; Myxococcales	GIMI china
*Myxococcus virescens*	GIM1.681	Bacteria; Proteobacteria; Deltaproteobacteria; Myxococcales	GIMI china
*Pyxidicoccus fallax*	GIM1.678	Bacteria; Proteobacteria; Deltaproteobacteria; Myxococcales	GIMI china
*Myxococcus stipitatus*	GIM1.683	Bacteria; Proteobacteria; Deltaproteobacteria; Myxococcales	GIMI china
*Corallococcus exiguus*	GIM1.684	Bacteria; Proteobacteria; Deltaproteobacteria; Myxococcales	GIMI china
*Cystobacter violaceus*	GIM1.680	Bacteria; Proteobacteria; Deltaproteobacteria; Myxococcales	GIMI china
*Cystobacter minus*	GIM1.679	Bacteria; Proteobacteria; Deltaproteobacteria; Myxococcales	GIMI china
*Archangium* sp.	GIM1.677	Bacteria; Proteobacteria; Deltaproteobacteria; Myxococcales	GIMI china
*Archangium gephyra*	DSM 2261	Bacteria; Proteobacteria; Deltaproteobacteria; Myxococcales	DSMZ Germany
*Corallococcus macrosporus*	DSM 14697	Bacteria; Proteobacteria; Deltaproteobacteria; Myxococcales	DSMZ Germany
*Corallococcus coralloides*	DSM 52499	Bacteria; Proteobacteria; Deltaproteobacteria; Myxococcales	DSMZ Germany
*Myxococcus fulvus*	DSM 16525	Bacteria; Proteobacteria; Deltaproteobacteria; Myxococcales	DSMZ Germany
*Myxococcus virescens*	DSM 2260	Bacteria; Proteobacteria; Deltaproteobacteria; Myxococcales	DSMZ Germany
*Cystobacter minus*	DSM 14751	Bacteria; Proteobacteria; Deltaproteobacteria; Myxococcales	DSMZ Germany
*Stigmatella erecta*	DSM 16858	Bacteria; Proteobacteria; Deltaproteobacteria; Myxococcales	DSMZ Germany
*Byssovorax cruenta*	DSM 14533	Bacteria; Proteobacteria; Deltaproteobacteria; Myxococcales	DSMZ Germany
*Other bacteria*			
*Chryseobacterium soli*	GIM1.315	Bacteroidetes; Flavobacteriia; Flavobacteriale;	GIMI china
*Burkholderia cepacia*	GIM1.139	Proteobacteria; Betaproteobacteria; Burkholderiales;	GIMI china
*Pediococcus acidilactici*	GIM1.263	Firmicutes; Bacilli; Lactobacillales	GIMI china
*Lactobacillus plantarum*	GIM1.380	Firmicutes; Bacilli; Lactobacillales	GIMI china
*Alcaligenes faecalis*	GIM1.61	Proteobacteria; Betaproteobacteria; Burkholderiales	GIMI china
*Salmonella paratyphi-A*	GIM1.235	Proteobacteria; Gammaproteobacteria; Enterobacteriales;	GIMI china
*Corynebacterium glutamicum*	GIM1.297	Actinobacteria; Actinobacteridae; Actinomycetales;	GIMI china
*Cellulomonas uda*	GIM1.188	Actinobacteria; Actinobacteridae; Actinomycetales;	GIMI china
*Microbacterium imperiale*	GIM1.280	Actinobacteria; Actinobacteridae; Actinomycetales;	GIMI china
*Sporolactobacillus inulinus*	GIM1.419	Firmicutes; Bacilli; Bacillales;	GIMI china
*Sphingobium amiense*	GIM1.502	Proteobacteria; Alphaproteobacteria; Sphingomonadales;	GIMI china
*Dokdonellafugitiva*	GIM1.474	Proteobacteria; Gammaproteobacteria; Xanthomonadales;	GIMI china
*Georgenia muralis*	GIM1.489	Actinobacteria; Actinobacteridae; Actinomycetales;	GIMI china
*Sphingomonas fennica*	GIM1.503	Proteobacteria; Alphaproteobacteria; Sphingomonadales;	GIMI china
*Bacillus megaterium*	GIM1.282	Firmicutes; Bacilli; Bacillales;	GIMI china
*Lactobacillus casei*	GIM1.159	Firmicutes; Bacilli; Lactobacillales;	GIMI china
*Escherichia coli*	GIM1.355	Proteobacteria; Gammaproteobacteria; Enterobacteriales;	GIMI china

a DSMZ, German collection of microorganisms and cell cultures; GIMI, Guangdong Institute of Microbiology.

### Primers design

Thirteen *mglA* gene sequences from identified species in seven different myxobacterial genera were retrieved from the GenBank, and aligned using ClustalW multiple sequence alignment program [16]. The aligned sequences included *Myxococcus xanthus* (AY197569), *Myxococcus Xanthus* DK1622 (CP000113), *Myxococcus xanthus* (AF377950), *Myxococcus fulvus* (CP002830), *Corallococcus coralloides* (CP003389), *Stigmatella aurantiaca* (CP002271), *Anaeromyxobacter dehalogenans* (CP001359), *Anaeromyxobacter* sp. (CP001131), *Anaeromyxobacter dehalogenans* 2CPC (CP000251), *Anaeromyxobacter* sp. (CP000769), *Polyangium cellulosum* (AY380815), *Sorangium cellulosum* (AM746676), *Haliangium ochraceum* (CP001804). The selected primers (Table 2) in this study were commercially synthesized (Guangzhou, China). Their specificity was assessed by using Primer-Blast (http://www.ncbi.nlm.nih.gov/) and tested by PCR amplification of DNA extracted from 17 myxobacterial strains as well as 17 non-myxobacterial strains. The optimal conditions for PCR reaction were evaluated by amplification from pure cultures of myxobacteria.

**Table 2:** PCR primers used in this study.

Primer sequences (5′→3′)	Tm values	Position^a^	length
*mglA*1F- CGCGAAATCAACTGCAAGAT	51.84	25∼44	20
*mglA2F*- CAGGTGTTCTACGACGCCA	53.43	244∼262	19
*mglA1R*-GGCAGGTCGCGCTTGTTGTACTG	60.83	415∼437	23
GC-*mglA*2F-CGCCCGCCGCGCGCGGCGGGCGGGGCGGGGGCACGGGGGGCAGGTGTTCTACGACGCCA	95.9	244∼262	59

a Correspond to the sequence number of gene *mglA* in *Myxococcus xanthus* 1622 (CP000113.1) [28].

### DNA extraction and purification

The pure cultures of myxobacterial strains were incubated on VY/2 medium for 3∼5 days at 30°C. The pure cultures of non-myxobacterial strains were cultivated in Luria-Bertani (LB) broth overnight at 37°C. The cells were collected and washed twice with sterile distilled water. DNA extraction from the pure cultures was performed as described by Murray and Thompson [17].

The soil metagenomic DNA extraction was carried out using a modified Bead-Beating method from Yeates and Gillings [18]. Briefly, 10 g of soil was suspended in 15 ml of extraction buffer [100 ml of 100 mM Tris-HCl (pH 8.0), 100 mM sodium EDTA (pH 8.0), 1.5 M NaCl]. Glass beads were added and the mixture was incubated at 37°C for 30 min with slow shaking. Sodium dodecyl-sulphate was added (2 ml, 20%, wt/vol) and blending continued for 10 min. The sample was transferred to a 65°C water bath for further 1 h incubation. During the incubation, the tube with mixture was reversed up and down at intervals to mix completely. The mixture was then centrifuged at 6,000 g for 15 min to remove soil residue. The supernatant was transferred to a clean tube (50 ml), and then precipitated by adding half-volume of polyethylene glycol (30%, wt/vol)/NaCl (1.6 M), and incubated at room temperature for another 2 h. The sample was centrifuged at 10,000 g for 20 min and the partially purified nucleic acid pellet was resuspended in 2 ml of TE (10 mM Tris-HCl, 1 mM sodium EDTA, pH 8.0). The crude metagenomic DNA extract was further purified by using equal volume of phenol/chloroform/isoamyl alcohol (25∶24∶1). Subsequently the metagenomic DNA was precipitated with 0.1 volume NaCl (3 M) and 0.6 volume of isopropanol. After 30 min at 20°C, DNA pellet was recovered by centrifugation at 10,000 g for 15 min. DNA pellet was washed by 70% (vol/vol) ethanol, air-dried and dissolved in 1 ml TE. The extracted DNA was then further purified with the Wizard Genomic DNA Purification Kit (Promega, USA).

### PCR amplification

All the PCRs were carried out in 25 µL reaction mixture contained 2.5 µL of 10×PCR buffer (plus Magnesiumion), 200 µM of dNTPs, 400 nM of each primer, 1.5 units of TaqDNA polymerase, and approximately 100 ng of template DNA per reaction. Eubacterial 16S rRNA was amplified using the primer sets 27F: 5′-AGAGTTTGATCCTGGCTCAG-3′ and 1492R: 5′-TACCTTGTTACGACTT-3′ [19], the following cycling protocol was used: one cycle of initial denaturation (94°C for 5 min), 35 cycles of denaturation (94°C for 40 s), annealing (56°C for 40 s) and extension (72°C for 1.5 min), followed by a final cycle of extension (72°C for 10 min).

The *mglA* gene was amplified from the DNA of the pure cultures and from the soil samples using myxobacteria-specific primers (Table 2). A semi-nested PCR approach was used to amplify *mglA* gene. The *mglA*1F/*mglA*1R primers were used in the first round of amplification, which were designed to obtain an 413 bp *mglA* gene fragment (the complete length of the *mglA* gene is 588 bp), and the GC-*mglA*2F/*mglA*1R primers were used for the second round, which were designed to amplify an approximate 194 bp *mglA* gene fragment. The first PCR reactions were carried out as follows: 5 min initial denaturation of DNA at 95°C, followed by 35 cycles of 40 s denaturation at 94°C, 40 s primer annealing at 58°C, and 1 min extension at 72°C, amplification was completed by a final extension step at 72°C for 10 min.

The first round PCR amplicons were diluted (1∶100) and a 1 µL dilution was used as template for the second round with GC-*mglA*2F/*mglA*1R primers. To increase the specificity of the second round amplification, a ‘touchdown’ PCR was performed under the following conditions: 10 cycles consisting of denaturation at 94°C for 30 s, annealing at 65°C∼55°C (the temperature was decreased by 1°C between consecutive steps) for 1 min and the extension at 72°C for 1 min and then 25 additional cycles consisting of denaturation at 94°C for 30 s, annealing at 55°C for 30 s and extension at 72°C for 1 min, amplification was completed by a final extension step at 72°C for 10 min. Amplified DNA fragments were checked by electrophoresis in 1.0% agarose gel. The products of the second round were used for DGGE analysis. When necessary, products were stored at −20°C before using.

### Denaturing gradient gel electrophoresis analysis

Analyses of the PCR amplicons were performed on using The DCode Universal Mutation Detection System (Bio-Rad) according to the protocol of Muyzer et al [11]. PCR products were loaded directly onto 8% (wt/vol) polyacrylamide gels in 1×TAE buffer (20 mM Tris-acetate [pH 7.4], 10 mM sodium acetate, 0.5 mM disodium EDTA) with gradients which were formed with 8% (wt/vol) acrylamide stock solution (acrylamide: bis-acrylamide, 37.5∶1). The denaturing gradient contained 35∼70% denaturant [100% denaturant corresponded to 7 M urea and 40% (vol/vol) deionized formamide]. Electrophoresis was performed at a constant voltage of 70 V for 12 h and a temperature of 60°C. After electrophoresis, the gel was stained with ethidium bromide (0.5 mg/ml), rinsed for 10 min with sterile Milli-Q water. The bands were visualized on a UV transillumination (302 nm) table equipped with a digital CCD camera.

### Sequencing and phylogenetic analysis

Twenty-eight bands were excised from the DGGE gels with a surgical knife and were suspended into 1.5 ml sterile Eppendorf tubes. To elute the DNA, 50 µl of sterile TE buffer (10 mM Tris-HCl, 1 mM sodium EDTA, pH 8.0) was added and the DNA was kept at 4°C overnight. Two microlitres of the supernatant was used as template DNA in a PCR (50 microlitres) with the primers *mglA*2F (without GC clamp) and *mglA*1R as described above. The PCR products were retrieved and purified using a sanPrep column PCR product purification kit (Shanghai, China), according to the manufacturer's instruction. To obtain full length sequences of the bands, cloning was applied. All retrieved bands were re-amplified, ligated to the pCR 2.1 vector and transformed in *E. coli* JM109 competent cells using the original TA cloning vector kit (pCR 2.1 vector for *E. coli*: JM109, Invitrogen) according to the manufacturer's instructions. Sequencing was performed in Invitrogen Trading (Shanghai, china). The sequences have been submitted to the GenBank database.

The *mglA* gene sequences of soil samples were performed for BLAST search against the GenBank database to obtain the nearest related sequences. All the sequences as well as recognized *mglA* gene sequences of myxobacteria from GenBank, and *mglA* gene sequences from several type myxobacterial strains were obtained to analyze the phylogenic relationship of the sequences. Sequences were aligned with ClustalW program [16], and then corrected by manual adjustion. The same parts of all reference *mglA* gene sequences, approximately 194 bp in length, were selected for construction of a phylogenetic tree after alignment with our soil sequences. A phylogenetic tree was constructed using the neighbour-joining method with a gap penalty of 100%. The bootstrapping supports for the phylogenetic tree were calculated from a sample of 1000 times.

### Nucleotide accession numbers

All the *mglA* gene sequences in this study have been deposited in the GenBank nucleotide sequence database under accession numbers from KF597855 to KF597882 (clones of 194 bp fragments).

## Results

### Primer specificity

A comparison of thirteen *mglA* gene sequences representing all identified myxobacteria from GenBank were aligned using Clustal W program version 1.8 [16], and results suggested that three regions were potentially taxon-specific for myxobacteria. Searches for sequence similarity were then performed with the GenBank nucleic acid databases by using the BLAST algorithm. Meanwhile, the sequences were evaluated by Primer Primier5.0 software package [20]. Given the size and taxon specificity of the PCR products, two sets of primer pairs *mglA*1F/*mglA*1R (PCR size of 194 bp) and *mglA*2F/*mglA*1R (PCR size of 413 bp) were selected as potential taxon-specific primers to myxobacteria (Table 2).

**Figure 1:**
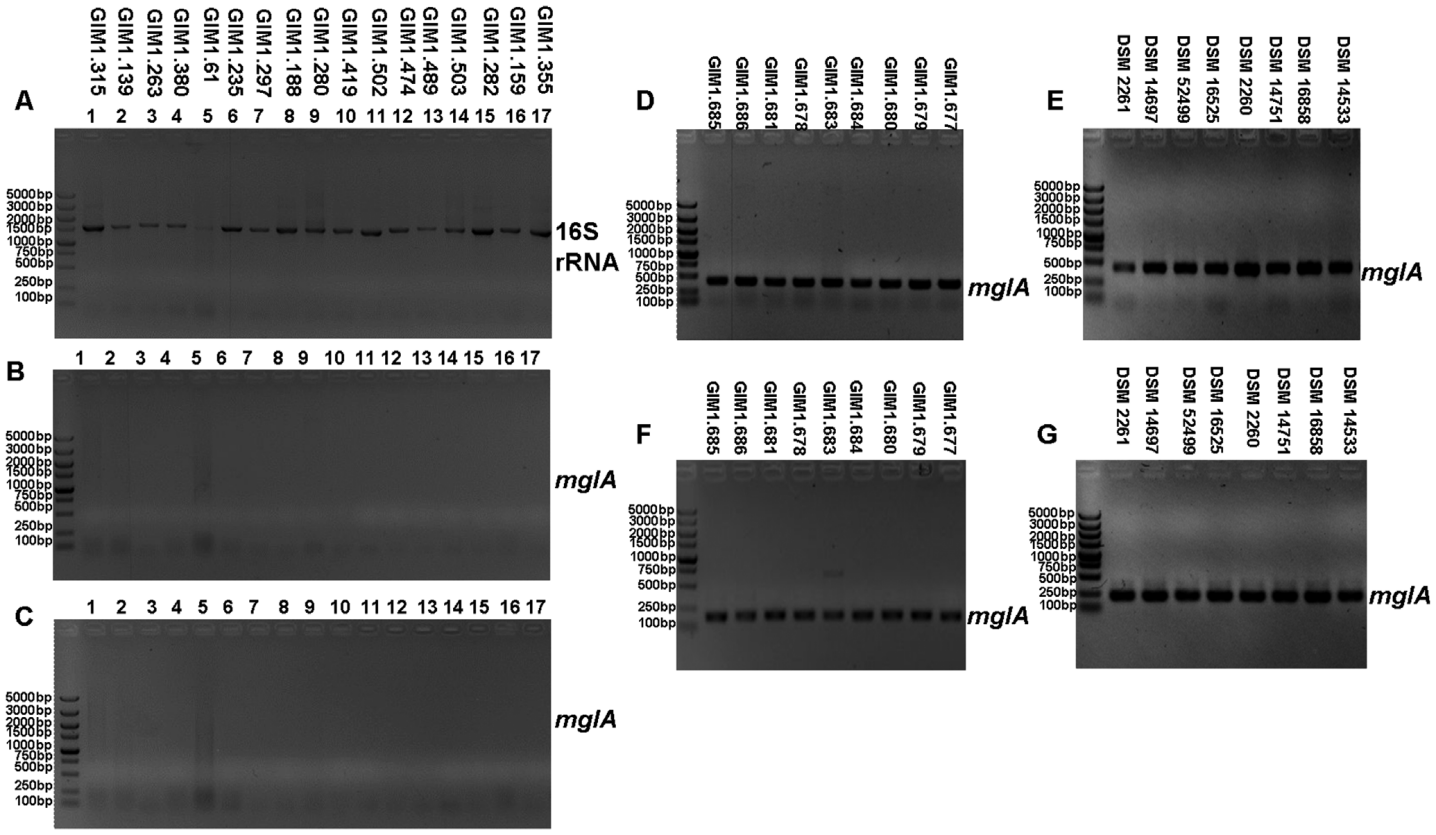
PCR amplifications of 16S rRNA with eubacterial and myxobacteria-specific primers. A. Primer 27F/1492R; B, D and E. Primer *mglA*1F/*mglA*1R; C, F and G. Primer *mglA*2F/*mglA*1R.

Specificity of the primer sets was evaluated using genomic DNAs from pure cultures of myxobacteria. Firstly, except for the quality of genomic DNA (Fig. 1A), no amplification was observed for seventeen non-myxobacterial strains (Table 1) using the same set of primers (Fig. 1B and 1C). And nine strains of myxobacteria obtained from GIMCC and eight from DSMZ (Table 1) were successfully amplified with expected size PCR product using primer pair *mglA*1F/*mglA*1R (Fig. 1D and 1E, 194 bp) and *mglA*2F/*mglA*1R (Fig. 1F and 1G), respectively. Thus, the two sets of primers are both especially taxon-specific, and strongly recommended as a useful gene marker for further study in myxobacteria.

### PCR-DGGE analyses of pure myxobacterial isolates

**Figure 2:**
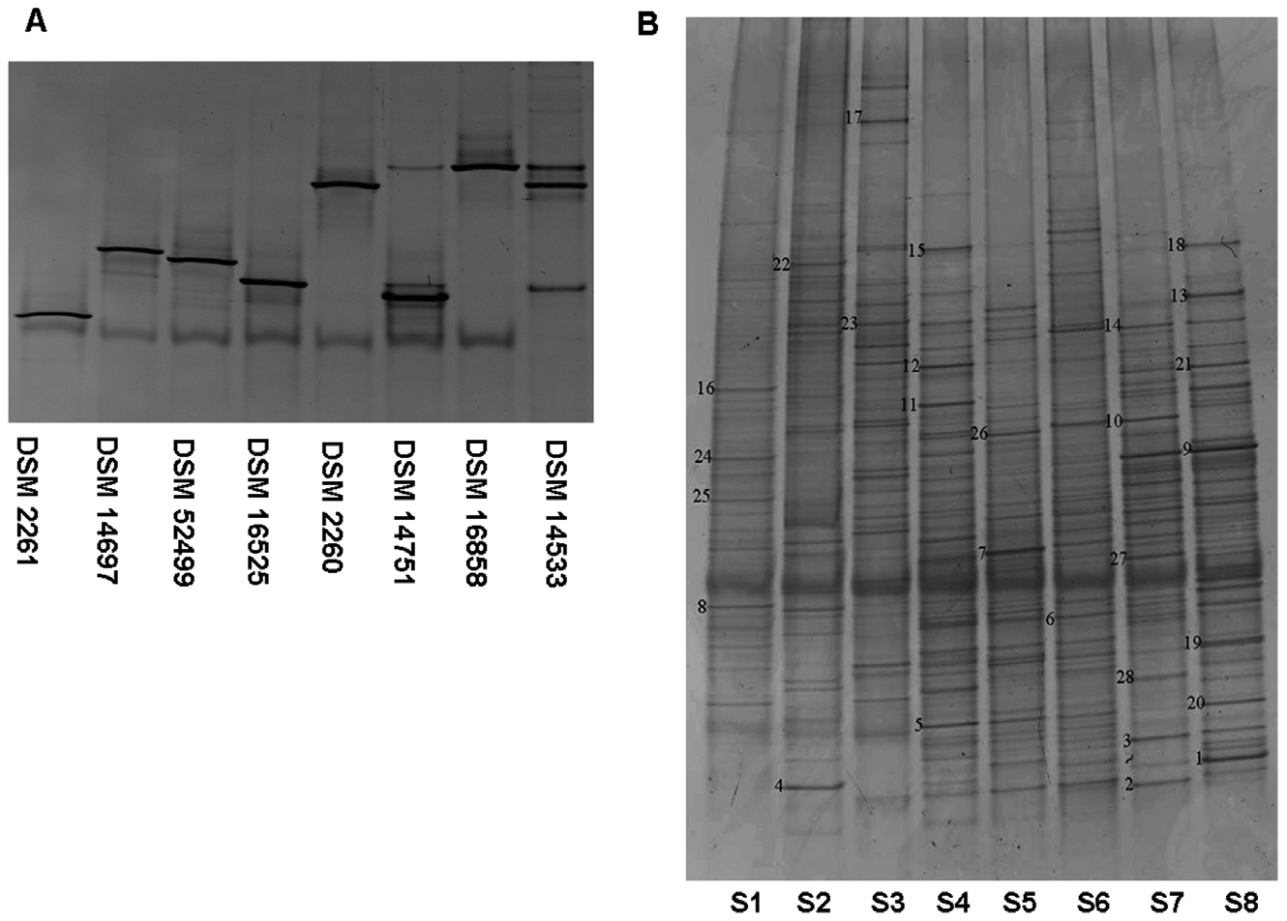
DGGE analyses of *mglA* gene fragments performed using DNA samples extracted from pure myxobacteria cultures (A) and soil samples (B). A lane, 1, *Archangium gephyra* DSM 2261; lane 2, *Corallococcus macrospores* DSM 14697; lane 3, *Corallococcus coralloides* DSM 52499; lane 4, *Myxococcus fulvus* DSM 16525; lane 5, *Myxococcus virescens* DSM 2260; lane 6, *Cystobacter minus* DSM 14751; lane 7, *Stigmatella erecta* DSM 16858; lane 8, *Byssovorax cruenta* DSM 14533; B, Bands (1∼28) that were excised for sequence analysis are numbered.

To analyze the diversity of *mglA* gene sequences in different myxobacterial strains with the taxon-specific primers, DGGE profiles of the *mglA* gene sequences were compared. Based on the DGGE profiles of six different pure myxobacterial strains (Fig. 2A), the optimal electrophoresis conditions were determined to be 14 h at 70 V with the denaturant gradient from 35% to 70%. The theoretical discrepancy characteristics of the sequences from above six tested myxobacteria strains were quite notable. PCR products with approximately 194 bp length were amplified using the primers GC-*mglA*2F/*mglA*1R (second-round PCR). As shown in Fig. 2A, PCR-DGGE profiles showed that there were sufficient differences in the migration of the amplicons to discriminate among the six tested myxobacterial species. The results of PCR-DGGE were in line with theoretical discrepancy.

### Myxobacteria diversity analysis using a semi-nested-PCR-DGGE strategy

The *mglA* gene, encoding for the gliding motility protein MglA, was confirmed to be conserved in the above results, which was consistent with previous study [14]. Therefore, the *mglA* gene could be selected as a candidate for microbial community analysis based on PCR-DGGE approach. Eight soil samples were selected to determine the putative diversity of myxobacteria and the DGGE analyses were performed with directly extracted DNA. To obtain more specific and accurate sequences data, semi-nest PCR was applied. The first-round PCR products of approximately 413 bp were amplified using the primers *mglA*1F/*mglA1R* and the PCR products were used as template of the second-round PCR. The second-round PCR products of approximately 194 bp were amplified using the primers *mglA2F*/*mglA1R* with GC clamps (Table 3). DGGE analyses were performed using the second-round amplicons to evaluate the myxobacteria diversity in soils (described in Experimental procedures). The DGGE profiles showed various banding patterns in different soil samples (Fig. 2B). A large number of bands were observed in the DGGE profiles of each samples, indicating highly diverse myxobacteria in soils. Some bands were shown specific to different soil samples, while others were commonly shared.

**Table 3:** Sequence similarity of excised DNA fragments.

Closest match	Band no.^a^ (Similarity)
*Anaeromyxobacter dehalogenans*	B15 (85%), B24 (90%)
*Anaeromyxobacter* sp.	B2 (75%)
*Anaeromyxobacter* sp. K	B28 (81%)
*Corallococcus coralloides*	B3 (90%), B6 (77%), B8 (88%), B10 (83%), B11 (81%), B17 (94%), B19 (85%), B26 (84%),
*Deinococcus maricopensis*	B1 (90%), B4 (91%), B27 (91%)
*Myxococcus fulvus*	B9 (97%), B14 (97%), B20 (97%), B21 (97%)
*Myxococcus xanthus*	B12 (99%), B16 (83%)
*Polyangium cellulosum*	B22 (90%)
*Stigmatella aurantiaca*	B5 (80%), B7 (87%), B13 (80%), B18 (84%), B23 (84%), B25 (96%)

a Bands B1 to B28 are the same bands as 1 to 28 in the denaturing gradient gel (Fig. 2B).

**Figure 3:**
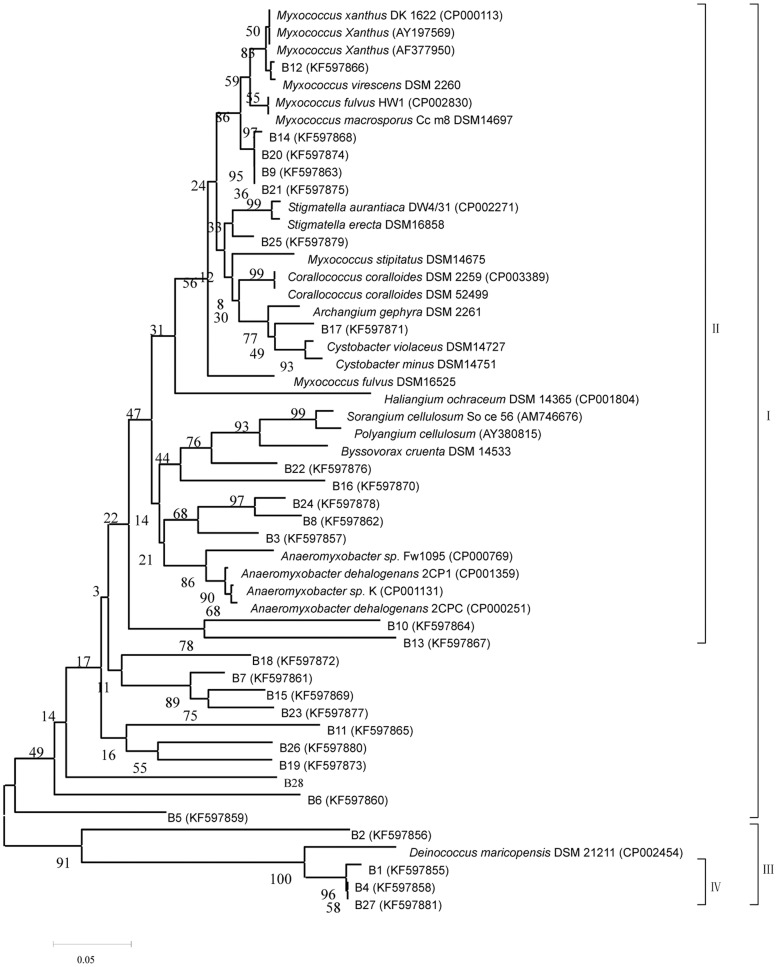
Phylogenetic tree showing the relationship of the twenty-eight retrieved bands from DGGE profile, on the basis of *mglA* gene fragment sequences obtained. The numbers of the sequences in this tree (e.g., B1) refer to the numbers in the denaturing gradient gel (i.e., 1 [Fig. 2B]). The tree was constructed using the neighbor-joining method and analysis was based on 194 nucleotides. Numerals at nodes indicate bootstrap values derived from 1000 replications [29].

In order to confirm the phylogenetic diversity of the amplified *mglA* gene fragment, twenty-eight dominant bands in different soil samples were recovered and sequenced (sequenced bands marked in Fig.2B) with phylogenetic affiliations represented in Fig. 3. The recovered bands were re-amplified, cloned and sequenced. The closest matches (and percentages of similarity) of the sequences were determined by a BLAST search. The results were shown in table 3.

According to the DGGE profiles, the majority (25 out of 28) of retrieved bands were closely related to myxobacteria including *Corallococcus*, *Anaeromyxobacter*, *Stigmatella*, *Myxococcus* and *Polyangium* and only three (band 1, 4, and 27) were clustered into the non-myxobacteria genus *Deinococcus* suggesting a portion homologous sequence shared by a few members of *Deinococcus*. These sequences were clustered into a single branch in the phylogenetic tree (clustal IV). The sequences similarities derived from bands 9, 12, 14, 17, 20, 21, and 25, were more than 94% to the sequences of three known genera of *Stigmatella*, *Myxococcus* and *Corallococcus*, and all of these bands clustered together into a big branch (clustal II). Another seven bands (3, 8, 10, 13, 16, 22 and 24) shared the same branch with former seven bands, which related to recognized myxobacteria of seven genus (submitted its recognized *mglA* gene sequences to GenBank), but sequences exhibited lower than 94% similarity with the myxobacteria sequences. There is no doubt that the above mentioned fourteen bands were derived from the members of myxobacteria. As shown in Fig. 3, the sequences derived from the bands (2, 5, 6, 7, 11, 15, 18, 19, 23, 26 and 28) were all closely related to myxobacteria, in spite of clustered into multiple branchs and low similarities with know myxobacteria, because of insufficiency sequences information of the *mglA* gene of myxobacteria. What's more, band 2 and band 5 were hard to make a taxon, which shared clustal III with non-myxobacterial strains, although the sequences were more related to myxobacteria.

## Discussion

As soil inhabitants, myxobacteria displayed complex life cycles, including swarming, fruiting body formation, and sporulation [1]. The species in this family exhibited gliding motility on solid surface in the absent of flagella, and the motility facilitates colony growth and expansion, as well as a wide range of behaviors such as predation, fruiting body formation and development [21], [22]. Furthermore, the mechanisms of gliding motility have remained unclear [22]. Previous works have showed that gliding motility was controlled by at least 37 genes, and the only one gene is indispensible to motility [14], [23]. Hartzell and Kaiser found that the MglA protein was highly conserved among myxobacteria [14]. In this study, we designed two pairs of primers based on the conserved regions of the *mglA* gene, and the specificity of primers was verified by PCR amplification. Our results showed that the designed primers were highly specific to myxobacteria, and conserved, in accordance with the results of MglA protein.

Myxobacteria are found nearly everywhere, from Antarctica to the tropics, and from water bodies to terrestrial [5]. The most diverse myxobacteria spp. were found in warm, semiarid areas and normally, a single spoonful of such soil might yield 5∼10 myxobacterial species. However, the ecological distribution of myxobacteria in different niches is still unknown. Our knowledge about myxobacteria diversity is mainly based on the traditional culture-dependent method [1], [5], however, there still did not have a highly efficient method for myxobacterial isolation, and inevitably underestimated the diversity of myxobacteria. Microbial communities in different environment have been frequently studied by using molecular ecological techniques, and DGGE has been generally adopt for its rapid, inexpensive and reproductive characteristics. Although Zhang et al [24] found that myxobacteria accounted for less than 1% of the total bacteria by the method of hybridization, the quantity and species of myxobacteria in complicated community are still unknown. This paper introduced a fast and economical DGGE technique to study the diversity of myxobacteria in complex microbial communities, however, the vital point is to find the myxobacteria-specific primers based on the specific and conserved genes of myxobacteria. Finally, we selected the *mglA* gene as target and two sets of primers were designed to amplify a portion of the *mglA* gene. The specificity of the primers were assessed by using the GenBank Basic Local Alignment Search Tool (BLAST) algorithm, and then DNAs extracted from seventeen bacteria (non-myxobacteria) and seventeen myxobacteria pure cultures were also used to verify the specificity of the designed primers. No amplification was obtained from all seventeen non-myxobacteria, meanwhile successful amplifications with expected size in length were generated from seventeen myxobacteria. All above results showed the designed primers based on the *mglA* gene were highly specific to myxobacteria and could be used as a molecular marker for study the myxobacteria diversity in different environment.

In this study, myxobacteria isolates belonging to six genus and eight species were distinguished by PCR combined with DGGE and different myxobacterial isolates generated various bands with different electrophoretic mobility distance in the DGGE profiles. All the myxobacterial isolates migrated to unique positions and could be easily distinguished from one another. The myxobacteria-specific marker in the *mglA* gene was proved to be useful to separate different myxobacterial isolates.

The method described in this study was proved to be highly effective in distinguishing pure cultures of myxobacterial isolates, however, the researchers usually focused more on the myxobacteria in different environmental samples, i.e. soil. Thus, myxobacteria diversity in eight soil samples were investigated by DGGE analyses based upon the *mglA* gene via semi-nested PCR. Our results clearly showed a number of visible bands in DGGE gel, implicating abundant myxobacterial resources in soils and the numbers of myxobacterial stains far more than Dawid's study, which only 1∼10 myxobacterial species could be found in a soil sample by analysis of 1398 soil samples from 64 countries in all continents [5]. Our results also suggested that there still many viable but uncultured myxobacterial strains undercurrent laboratory conditions. So we can assure that new myxobacterial resources may depend on new isolation methods, and new environment. Twenty-eight bands were selected, retrieved and sequenced. According to the above results, the similarities to recognized myxobacteria were generally low as the sequences of *mglA* gene related to myxobacteria in GenBank were limited to only thirteen strains.

Despite the high sensitivity of this technique, cases of comigration of distinct strains have been previously reported in other studies about PCR-DGGE [25], [26]. DGGE, theoretically, can detect down to one nucleotide variation [11], but separation of DNA fragments is influenced by the nucleotide composition and the length of nucleotide [26]. To overcome this limitation, the band should be excised, cloned into vectors and sequenced.

Moreover, some difficulties for an accurate identification of some myxobacteria species based on the analyses of 16S rRNA gene sequences and morphology approach [27], still remained to be resolved. The previous and our studies both confirmed that the *mglA* gene is conserved in myxobacteria species, and we could found that the reference strains of the same genus or species [27], which have studied its phylogenetic tree including all recognized genus based on 16S rRNA in Fig. 3, also clustered into the same clade. Therefore, it is highly recommended to combine 16S rRNA sequence and other myxobacteria-specific as well as conserved *mglA* gene analysis in order to identify a myxobacterial strain. We also hope that the *mglA* gene database should be improved.
